# Effectiveness of personalized treatment stage-adjusted digital therapeutics in colorectal cancer: a randomized controlled trial

**DOI:** 10.1186/s12885-023-10728-2

**Published:** 2023-04-03

**Authors:** Inah Kim, Ji Young Lim, Sun Woo Kim, Dong Wook Shin, Hee Cheol Kim, Yoon Ah Park, Yoon Suk Lee, Jung-Myun Kwak, Seok Ho Kang, Ji Youl Lee, Ji Hye Hwang

**Affiliations:** 1 Department of Physical and Rehabilitation Medicine, Samsung Medical Center, Sungkyunkwan University School of Medicine, 81 Irwon-ro Gangnam-gu, 06351 Seoul, Republic of Korea; 2 Research Institute for Future Medicine, Samsung Medical Center, 06351 Seoul, Republic of Korea; 3 Department of Family Medicine/Supportive Care Center, Samsung Medical Center, Clinical Research Design and Evaluation, SAIHST, Sungkyunkwan University, 81 Irwon-Ro, Gangnam-gu, 06351 Seoul, Republic of Korea; 4 Department of Surgery, Samsung Medical Center, Sungkyunkwan University School of Medicine, 81 Irwon-ro, Gangnam-gu, 06351 Seoul, Republic of Korea; 5 Division of Colorectal Surgery, Department of Surgery, Seoul St. Mary’s Hospital, College of Medicine, The Catholic University of Korea, Seoul, Republic of Korea; 6 Division of Colorectal Surgery, Department of Surgery, Korea University Anam Hospital, Seoul, Republic of Korea; 7 Department of Urology, Korea University Anam Hospital, 02841 Seoul, Republic of Korea; 8 Department of Urology, Seoul St. Mary’s Hospital, College of Medicine, The Catholic University of Korea, 06591 Seoul, Republic of Korea

**Keywords:** Mobile health, Digital therapeutics, Colorectal cancer, Randomized Controlled Trial

## Abstract

**Background:**

Colorectal cancer survivors often experience decline in physical performance and poor quality of life after surgery and during adjuvant therapies. In these patients, preserving skeletal muscle mass and high-quality nourishment are essential to reduce postoperative complications and improve quality of life and cancer-specific survival. Digital therapeutics have emerged as an encouraging tool for cancer survivors. However, to the best of our knowledge, randomized clinical trials applying personalized mobile application and smart bands as a supportive tool to several colorectal patients remain to be conducted, intervening immediately after the surgical treatment.

**Methods:**

This study is a prospective, multi-center, single-blinded, two-armed, randomized controlled trial. The study aims to recruit 324 patients from three hospitals. Patients will be randomly allocated to two groups for one year of rehabilitation, starting immediately after the operation: a digital healthcare system rehabilitation (intervention) group and a conventional education-based rehabilitation (control) group. The primary objective of this protocol is to clarify the effect of digital healthcare system rehabilitation on skeletal muscle mass increment in patients with colorectal cancer. The secondary outcomes would be the improvement in quality of life measured by EORTC QLQ C30 and CR29, enhanced physical fitness level measured by grip strength test, 30-sec chair stand test and 2-min walk test, increased physical activity measured by IPAQ-SF, alleviated pain intensity, decreased severity of the LARS, weight, and fat mass. These measurements will be held on enrollment and at 1, 3, 6 and 12 months thereafter.

**Discussion:**

This study will compare the effect of personalized treatment stage-adjusted digital health interventions on immediate postoperative rehabilitation with that of conventional education-based rehabilitation in patients with colorectal cancer. This will be the first randomized clinical trial performing immediate postoperative rehabilitation in a large number of patients with colorectal cancer with a tailored digital health intervention, modified according to the treatment phase and patient condition. The study will add foundations for the application of comprehensive digital healthcare programs focusing on individuality in postoperative rehabilitation of patients with cancer.

**Trial registration:**

NCT05046756. Registered on 11 May 2021.

**Supplementary Information:**

The online version contains supplementary material available at 10.1186/s12885-023-10728-2.

## Background

Colorectal cancer is the third and second most common cancer worldwide in men and women, respectively [1]. Colorectal cancer survivors often suffer from decline in physical performance and mental health, psychological distress, and difficulty in returning to society after surgery and during adjuvant therapies. For example, 80–90% of individuals who undergo sphincter-preserving surgery experience lower anterior resection syndrome [1, 2]. Patients experience difficulties not only in the active treatment phase but also in expanded phases even after the completion of cancer treatment. Thus, physical fitness, quality of life, and social issues of colorectal patients should be addressed from both immediate and long-term perspectives. To be more specific, clinicians should be aware of the hardships the patients confront according to the characteristics of therapeutic phases and individuality of each patient for comprehensive management of the disease.

Health behaviors, such as physical activity and nutrition, can have positive effects on physical functioning, quality of life, and symptoms related to cancer treatment, such as fatigue among cancer survivors [3]. In fact, low muscle mass is frequently observed in patients with colorectal cancer, ranging between 12 and 60% [4]. It serves as a predictor of postoperative complications, chemotherapy toxicity, reduced quality of life, and decreased overall survival [4]. Previous studies have reported that exercise has the potential to reverse severe muscle loss in patients with cancer [5]. Furthermore, nutritional support such as adequate dietary intake and high protein is reported to contribute to muscle mass preservation [4]. Hence, a multimodal approach including nutritional intervention and fitness programs is essential in patients with colorectal cancer.

At present, many cancer survivors rarely use tools to report subjective data such as pain, fatigue, and distress [6]. Moreover, many patients face difficulties to receive sufficient and appropriate information applicable to their daily lives [7]. Thus, development of a comprehensive, easy-to-access platform for managing oncological outcomes, body composition, and functional status is becoming indispensable since their alterations may influence the prognosis of the disease [4].

Mobile health care application has emerged as an encouraging device in managing the clinical course of cancer and improving quality of life and physical performance in cancer survivors. Mobile health is defined as the provision of health services and information through mobile and wireless technologies [8]. A previous study showed that physical capacity including muscle strength of lower extremities and cardiorespiratory endurance and treatment-related symptoms, such as fatigue, nausea, and vomiting, were significantly improved in colorectal cancer patients through a tailored rehabilitation exercise program supported by mobile health applications [1]. However, generalization to overall colorectal cancer patients could not be done owing to a limited number of patients and confinement to a specific phase of the treatment.

To the best of our knowledge, randomized clinical trials that applied a personalized, phase-specific digital health program using mobile application and smart bands to a large number of patients with colorectal cancer have not yet been conducted, especially starting immediately post cancer operation. The primary objective of this protocol is to clarify the effect of mobile application and wearable smart band on skeletal muscle mass increment in patients with colorectal cancer. The secondary outcomes will be improvement in quality of life, enhanced physical fitness level, increased physical activity, diminished pain intensity, decreased severity of the low anterior resection syndrome, weight, and fat mass.

## Methods

### Study design

**Fig. 1:**
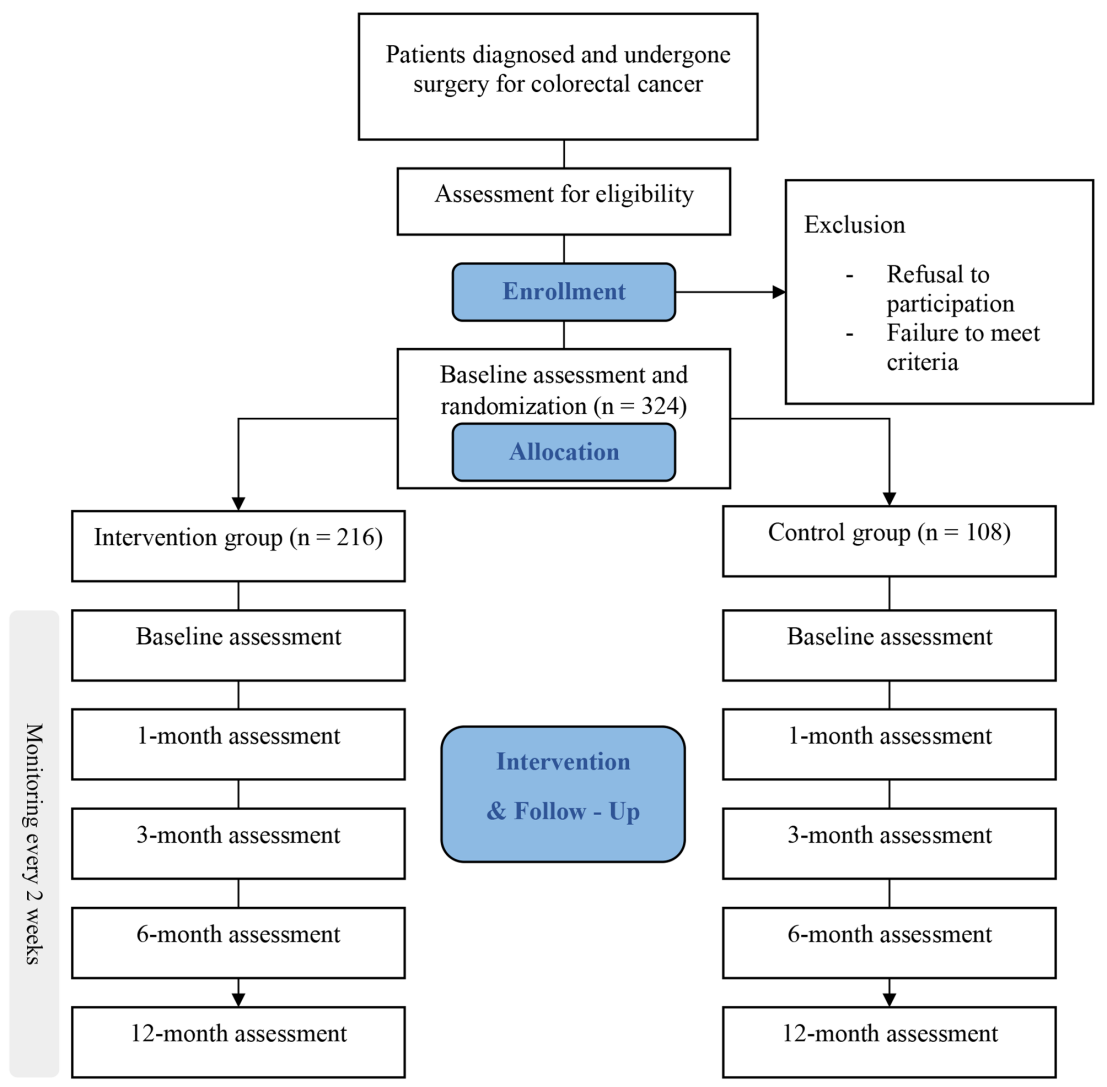
Flow chart of the randomized controlled trial

This protocol will be a prospective multi-centered two-armed randomized controlled trial. The study design meets SPIRIT guidelines [9]. The SPIRIT Checklist are attached as Additional file 1. Colorectal cancer patients will be recruited from three university hospitals in South Korea (Samsung Medical Center, Korea University Anam Hospital, and Seoul St. Mary’s Hospital). The enrolled participants will be randomly allocated to either the digital therapeutic or control group. Regardless of group allocation, all patients will receive conventional education and usual care in hospital. Patients who undergo colostomy will receive colostomy care by hospital staffs. Furthermore, patients who underwent low anterior resection surgery will receive education of the pelvic floor muscle rehabilitation by a physical therapist, 2–4 weeks after surgery. The flow diagram of the study protocol is shown in Fig. 1.

### Participants

In this study, we will enroll 324 participants who were diagnosed with colorectal cancer and have undergone surgery. The inclusion criteria will be as follows: (a) aged 19–85 years, (b) diagnosed with colorectal cancer and undergone colorectal cancer resection surgery, (c) uses an Android- or iOS-based smartphone, (d) able to use mobile application and have regular follow-up assessment as outpatient, (e) voluntary participation. The exclusion criteria will be as follows: (a) unable to perform exercise and diet management because of severe underlying disease, neuromusculoskeletal disease, or cognitive or visual impairment, (b) communication difficulties.

All eligible patients will be informed about this study, and after consultation with the department of surgery and the department of rehabilitation medicine they will be invited to participate. All patients will submit a written informed consent if they agree to participate in the study.

### Randomization, allocation, and blinding

We used blocked randomization with randomly selected block sizes (block size: 3 and 6). This study is an open study; therefore, the name of the group is not blinded. Owing to the nature of the intervention, the participant and evaluator can recognize which intervention has been assigned to the participant. After randomly allocating patients into two groups with a 1 (control group): 2 (intervention group) ratio, baseline assessment will be performed.

### Interventions

#### Personalized digital therapeutic (intervention) group

For the personalized digital therapeutic group, Colon Cancer by Second Doctor program (Medi Plus Solution, Seoul, Korea) for health management after colorectal cancer surgery and DoFit as a smart band on the wrist (NF-B20, Medi Plus Solution, Seoul, South Korea) will be used. The application and fundamental contents are developed by an expert team consisting of app developers, designers, service programmers, cancer rehabilitation specialists, and researchers as the basis of evidence. It can be used on both Android and iOS platforms. Data collected from smart band can be transferred to application via Bluetooth connection. This smart band is capable of measuring the physical activity (step counts and energy expenditure), heart rate through a built-in 6-axis accelerometer, gyroscope, and photoplethysmography sensor. In addition, information from smart devices such as blood pressure monitors, blood glucose monitors, and weight scales can be linked with the application.

**Fig. 2:**
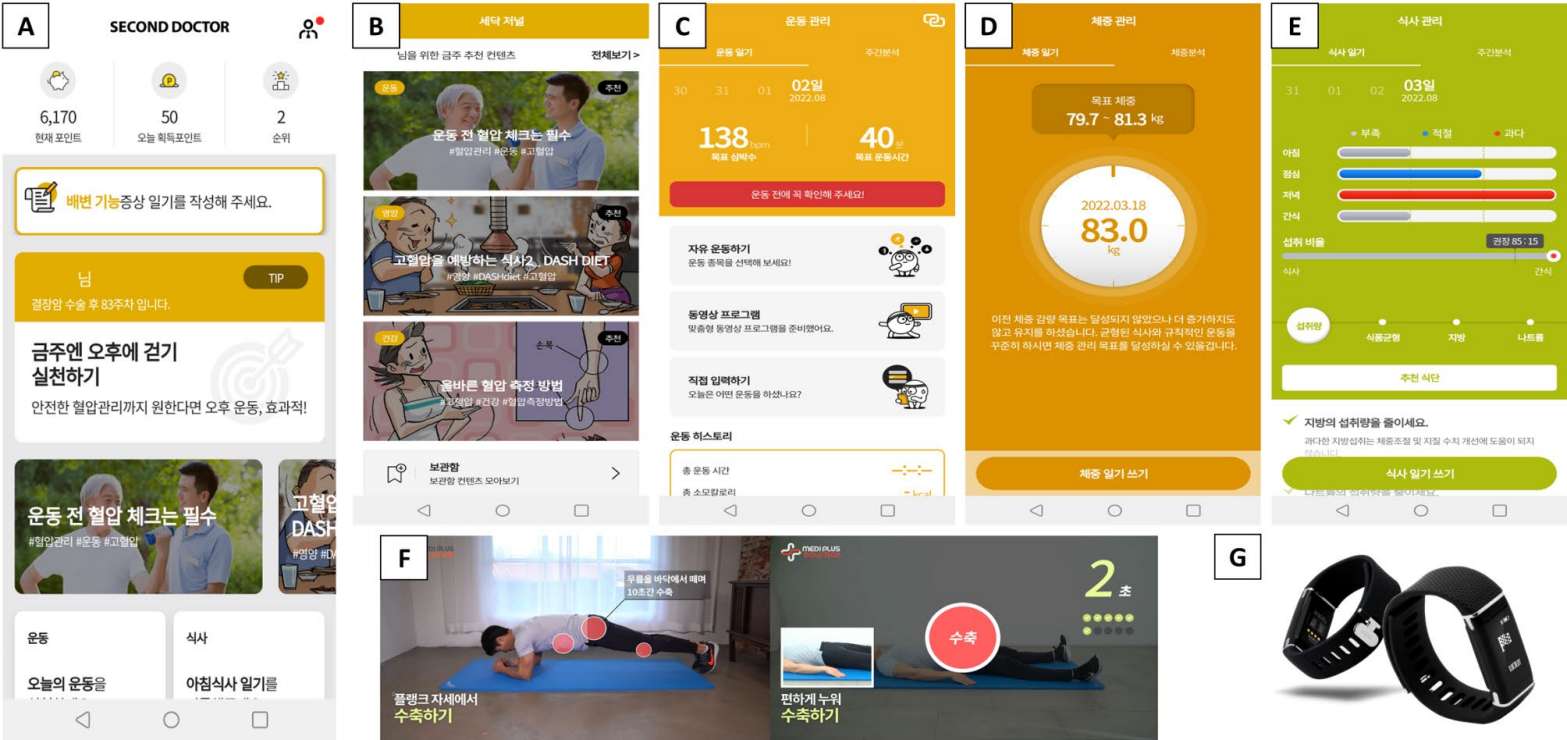
Screenshots of the representative function and smart-band: (A) home (today’s to do list), (B) second doctor journal, (C) exercise management, (D) weight management, (E) diet management, (F) exercise video (pelvic floor muscle training), and (G) DoFit. Reproduced with permission of Medi Plus Solution. Co., Ltd

The key app functions are indicated in Table 1 and screenshots of representative functions are shown in Fig. 2. User information for the algorithms of personalized content includes treatment type (chemotherapy), hypertension, initial weight, age, perceived rating of exertion, and comorbid diseases. After the primary analysis using these factors, the user life log data is repeatedly analyzed to provide a tailored guide.

Health care professionals can monitor the individual usage of participant using a web-based open architecture management program including all life log data. To manage the adherence to the mHealth-based intervention, we will monitor the application usage rate every 2 weeks for 12 months, and feedback will be provided during initial 3 months.

### Control group

The control group will go through conventional education and usual care in hospital for 12 months.

**Table 1:** Functions of the mobile application and key characteristics

Functions	Key characteristics
Expert consultation	• It provides expert consultations regarding exercise and nutrition using text messages, voice recordings, and images. It does not apply to consultations on symptoms. User can receive the result of consultation within 24 h.
Second doctor Journal	• It offers health information and education contents regarding exercise, nutrition, and disease according to the surgery, treatment type, and comorbidities.
Exercise management	• It offers an aerobic exercise program with target heart rate and exercise time according to the user’s treatment type. In addition, using a weekly step-by step approach, the exercise program with video clips is provided by combination of stretching and muscle strengthening exercises and pelvic floor muscle training. • The contents of exercise are personalized according to the user information (surgery and treatment type); difficulty is adjusted by rating the perceived exertion after the exercise.
Diet management	• If a user inputs their daily diet using speech recognition and text input functions, it provides feedback of the intake, food balance, and protein and fat, with the marking as insufficient, adequate, or excessive. • The weight information entered at the time of membership registration becomes the standard for the initial recommended calorie intake. • It simply recommends foods and its amount for breakfast, lunch, dinner, and snacks. Moreover, it recommends nutritional intake according to comorbid diseases from the 13th week.
Physical activity management	• It recommends the target stepping intensity, burning of calories, and heart rate (e.g., 5,000 steps, 200 kcal, etc.). The number of steps according to the step type is displayed.
Comorbidity and Weight management	• It indicates target blood pressure and blood glucose level based on the clinical practice guidelines. • It informs the user whether their weight is within the normal weight range.

### Outcome measures

Outcomes will be assessed at baseline and at 1-, 3-, 6-, and 12-month follow-ups. Outcome measures will be assessed by clinical research coordinator and summary of baseline screening, assessment, and follow-up during study visits is described in Table 2.

**Table 2:** Summary of baseline screening, assessment, and follow-up during study visits

		STUDY PERIOD				
		Post-allocation				
TIMEPOINT		*Post op* *(± 1w)*	*1-month* *(± 2w)*	*3-month* *(± 1 m)*	*6-month* *(± 2 m)*	*12-month* *(± 2 m)*
	**ENROLLMENT**:					
***1***	***Eligibility screening***	X				
***2***	***Informed consent***	X				
	***Allocation***	X				
***3***	***Demographic characteristic***	X				
***4***	***Medical history***	X				
***5***	***Health lifestyle***	X				
***6***	^***b***^ ***eHealth Literacy Scale***	X				
	**INTERVENTIONS**:					
	***Personalized digital therapeutic (intervention) group***					
	***Control group***					
	**ASSESSMENTS**:					
***7***	***Complication***		X	X	X	X
***8***	***Hb, albumin***	X ^1)^			X	X
***9***	***CT (visceral fat, muscle mass)***	X ^1)^			X	X
***10***	***Body composition***	X	X	X	X	X
***11***	***EORCT-QLQ-C30***	X	X	X	X	X
***12***	***EORCT-QLQ-CR29***	X	X	X	X	X
***13***	***MNA***	X	X	X	X	X
***14***	***IPAQ-SF***		X	X	X	X
***15***	***Grip strength***	X	X	X	X	X
***16***	***30 s chair stands test***		X	X	X	X
***17***	***2 min walk test***		X	X	X	X
***18***	^***a***^ ***LARS***		X	X	X	X
***19***	^***b***^ ***Satisfaction questionnaire***				X	

*CT*: computed tomography, *EORCT-QLQ*: European Organization for Research and Treatment of Cancer-Quality of life Questionnaire, *MNA*: Mini Nutritional Assessment, *IPAQ-SF*: International Physical Activity Questionnaire-Short Form, *LARS*: Low Anterior Resection Syndrome Score Questionnaire

^a^ Rectal cancer without ileostomy and colostomy.

^b^ Digital therapeutic group only.

^1)^ Tests analyzed within previous 6 months from the baseline can be collected.

### Primary outcome

The primary objective of this study is to determine the effect of mobile application and wearable smart band in the management of prognostic factors in colorectal cancer patients. Therefore, the primary outcome would be the skeletal muscle mass increment in the intervention group compared to the control group in 12 months from the baseline, measured using the bio impedance analysis.

### Secondary outcomes

The secondary objectives of our study are quality of life, body function and composition, and pain intensity over time.

The quality of life will be evaluated using the European Organization for Research and Treatment of Cancer-Quality of life Questionnaire (EORCT-QLQ-C30 and CR29). EORTC QLQ-C30 is composed of functional, symptom, and global quality of life domain. The quality-of-life scale uses a modified 7-point linear analog scale. All other items are scored on a 4-point categorical scale, ranging from 1 “not at all” to 4 “very much” [10]. The EORTC QLQ-CR29 is designed to complement the C30 questionnaire as a tumor-specific questionnaire for patients with colorectal cancer. It is composed of function and symptom domains [11, 12]. High overall quality-of-life, high functional domain score, and low symptom domain score correlates with high quality of life.

International physical activity questionnaire-short form (IPAQ-SF) is divided into vigorous activity, moderate activity, walking, and lying down to evaluate the amount of activity in the last seven days. The participants are also asked to define the number of min and days spent performing a specific activity category [13].

Mini nutritional assessment (MNA) defines nutritional status. It is divided into several domains for anthropometric assessment (weight, height, and weight and appetite loss) [14]. The total score is 30 points, < 17 points indicates malnutrition, 17–23.5 points indicate risk of malnutrition, and 24–30 points is considered normal.

Physical fitness is evaluated using the grip strength test, 30-sec chair stand test (CST), and 2-min walk test (2MWT). Grip strength is assessed using a hand-held dynamometer (microFET® Digital HandGRIP Dynamometer, Hoggan Scientific LLC, USA). The patients are asked to use the maximal hand grip power (kg). The patients repeat this three times for three seconds, and the average of the results will be recorded [15]. The CST is used to evaluate the strength of the lower extremities by assessing the maximal total number of repetitions of standing up to a chair that the person could achieve in 30 s. Participant is instructed to stand up and sit down fully at their maximal speed for 30 Sects. [16, 17]. The 2MWT is used to examine the cardiorespiratory endurance of the participants. It will be performed in a 15.2-m hallway. The patients will be asked to walk at their maximal speed for 2 min and the total distance they walked will be measured [18].

The abdomen and pelvis will be analyzed using computed tomography (CT). To measure muscle mass and visceral fat, we will analyze the cross-sectional area in a single CT slice at the height of the third lumbar vertebra [19].

Rectal cancer patients without ileostomy or colostomy would be evaluated for defecation function by Low Anterior Resection Syndrome Score (LARS) questionnaire. Patients are categorized into three groups: no LARS (0–20), minor LARS (21–29), and major LARS (30–42) [20, 21].

The pain intensity for the past week will be evaluated by a 11-point Numeric Rating Scale (NRS) [22] with 0 and 10 representing “no pain” and “the worst possible pain”, respectively. Participants will answer about an average and the worst perceived pain intensity score.

Additionally, the intervention group will undergo a general assessment with e-health literacy at baseline and the self-developed satisfaction questionnaire for mobile application and Internet of Things device after 6 months. The eHealth Literacy Scale (eHEALS) is designed to assess consumer’s perceived skills at using information technology for health and to aid in determining the applicability of eHealth programs for individual consumers. The eHEALS has 10 items, where items 1 and 2 are supplementary. Eight questions assess eHealth literacy on a 5-point Likert scale, in which a higher score indicates higher literacy [23].

### Sample size calculation

Based on a previous study [24], the sample size was calculated with effect size of 0.35 (moderate level), 80% of power, and allocation ratio 1 (control group): 2 (intervention group) using the G-power 3.1.9.2. The results showed that the number of intervention and control group participants were at least 195 and 97, respectively. Considering the drop-out rate (10%), total number of participants was 324 (216 of intervention group and 108 of control group). We will enroll competitively participants to reach target sample size.

### Data management

For systematic data management, electronic data capture of Medicallogic Company (www.medicallogic.com) will be used for this study. During the study, the electronic case report form for each enrolled participant will be filled in with all data, which includes the baseline visit, and clinical data, such as comorbidities. All relevant data will be uploaded from the source documents into an electronic case report form by study-site personnel. This web-based platform will be accessible for permitted research team members only.

### Statistical analysis

All data will be analyzed using IBM SPSS Statistics version 28.0.1 (Armonk, New York, USA) and a significance level of 5% will be set with a 95% confidence interval. Patient demographics will be calculated using descriptive statistics. Kolmogorov–Smirnov test will be performed to assess the distribution of data. To compare baselines between the two groups, a student’s t-test and chi-squared test will be conducted. In case the randomization is not balanced, the baseline difference will be corrected through multivariate logistic regression and analyzed. To compare the mean difference, if the primary outcome (skeletal muscle mass) data in both groups are found to be normally distributed, we will perform an independent t-test. Missing data will be handled depending on the distribution of data after study completion. To investigate the effects of the interventions and differences between groups for secondary outcomes, a mixed effects model or generalized estimating equations will be conducted, with one between-subject factor (group) and one within-subject factor (evaluation time).

## Discussion

Owing to COVID-19 pandemic, home-based exercise is emerging as a promising tool for physical therapy. However, the biggest shortcoming of home-based program is still the poor management of compliance. Contrarily, previous studies on health management service applications had segmental interventions, such as only applying the health program during chemotherapy or dealing mainly with only psychological or nutrition-related issues [25, 26]. Most of them enrolled a small number of patients; insufficient to apply to general population. Although, studies regarding digital health issues are increasing in number, cancer related issues have not been addressed yet. Until now, adequate home-based exercise program with digital health platform that patients with colorectal cancer can use has been lacking, especially for the patients not eligible for regular visit to outpatient clinic. Therefore, for the sake of such patients, a comprehensive multimodal personalized digital intervention with easy-to-access mobile app is necessary, which manages hardships in every postoperative phase and enhances the patients’ physical activity and nutrition right after the operation. Consequently, a good quality of life, enhanced physical activity, and an easy return-to-society can be achieved, by managing the health behavior and nutrition status of the patients.

To the best of our knowledge, this will be the first randomized clinical trial performing digital therapeutics for personalized postoperative rehabilitation in patients with colorectal cancer. The study has several differentiated strong points. First, it starts immediately after the operation for one year. Second, a large number of colorectal cancer patients will be enrolled. Third, the program will be composed of a tailored digital health intervention, modified according to the treatment phase and patient condition. Another short-term positive consequence of our program includes reduction of financial and time burden on patients as well as on clinicians. In the long term, an integrated medical management of disease and psychological intervention would enable more comprehensive disease controlling systems.

In conclusion, this study will clarify the effect of patient-centered mobile and wearable digital health interventions on immediate postoperative rehabilitation of the patients with colorectal cancer. The study will add foundations for the application of comprehensive digital healthcare programs in postoperative rehabilitation for cancer patients in remote areas.

### Trial status

The first participant recruitment began on 11 May 2020.

### Participant safety and withdrawal

The researcher’s name and phone number for an emergency contact will be provided to ensure the patient’s safety. All participants are informed that they can voluntarily discontinue the study at any time and they could be withdrawn when the significant disease non-related to study is detected, cancer metastasis and recurrence occurs, or if they did not follow the instruction of doctor in charge.

### Ethics and dissemination

All study procedures were approved by the Institute Review Board of three hospitals (approval numbers: SMC-2021-01-090, 2021AN0104 and KC21FNSI0177). The trial is registered on clinicaltrials.gov (approval ID: NCT05046756). The study protocol was reviewed by the institutional review board of Samsung Medical Center on 1 Feb 2021 and was approved on 23 Feb 2021 as original protocol. In the case of important protocol modifications, principal investigator will share them with coordinating investigators and trial participants, and report to the institutional review board.

Personal information about enrolled participants will be collected, shared with clinic (i.e., Korea University Anam Hospital and Seoul St. Mary’s Hospital), and retained only by the research team during study. After the end of the study, all personal information will be retained for 3 years and subsequently destroyed.

The trial results will be published in the journal and report of results will be posted on the funding institute’s site for the public, participants, and healthcare professionals.

Any publications containing the results of this study have not been already published or submitted to any journal.

## Electronic supplementary material

Below is the link to the electronic supplementary material.

**Additional files 1.** SPIRIT Checklist.

## Data Availability

Not applicable.

## List of Abbreviations

CT: Computed tomography
EORCT-QLQ: European Organization for Research and Treatment of Cancer-Quality of life Questionnaire
MNA: Mini Nutritional Assessment
IPAQ-SF: International Physical Activity Questionnaire-Short Form
LARS: Low Anterior Resection Syndrome
CST: 30-second chair stand test
2MWT: 2-minute walk test
NRS: Numeric Rating Scale
eHEALS: eHealth Literacy Scale

## References

[1] Cheong IY, An SY, Cha WC, Rha MY, Kim ST, Chang DK, Hwang JH (2018). Efficacy of Mobile Health Care Application and Wearable device in improvement of physical performance in Colorectal Cancer Patients undergoing chemotherapy. Clin Colorectal Cancer.

[2] Nguyen TH, Chokshi RV (2020). Low anterior resection syndrome. Curr Gastroenterol Rep.

[3] Speck RM, Courneya KS, Masse LC, Duval S, Schmitz KH (2010). An update of controlled physical activity trials in cancer survivors: a systematic review and meta-analysis. J Cancer Surviv.

[4] Vergara-Fernandez O, T-AM, Salgado-Nesme N (2020). Sarcopenia in patients with colorectal cancer: a comprehensive review. World J Clin Cases.

[5] Derksen JWG, Kurk SA, Oskam MJ, Peeters PHM, Punt CJA, Koopman M, May AM (2019). Factors contributing to Cancer-Related muscle wasting during first-line systemic treatment for metastatic colorectal Cancer. JNCI Cancer Spectr.

[6] Soh JY, Cha WC, Chang DK, Hwang JH, Kim K, Rha M, Kwon H (2018). Development and validation of a Multidisciplinary Mobile Care System for patients with Advanced Gastrointestinal Cancer: Interventional Observation Study. JMIR Mhealth Uhealth.

[7] Kim KS. Polarization of cancer patient management. Journal of the Korean Medical Association 2017, 60(3).

[8] Lewis J, Ray P, Liaw ST. Recent Worldwide Developments in eHealth and mHealth to more Effectively Manage Cancer and other Chronic Diseases - A Systematic Review. Yearb Med Inform 2016(1):93–108. 10.15265/IY-2016-020 PMC5171554 27830236

[9] Chan A-W, Tetzlaff JM, Gøtzsche PC, Altman DG, Mann H, Berlin JA, Dickersin K, Hróbjartsson A, Schulz KF, Parulekar WR. SPIRIT 2013 explanation and elaboration: guidance for protocols of clinical trials. Bmj 2013,346. 10.1136/bmj.e7586 PMC3541470 23303884

[10] Giesinger JM, Kieffer JM, Fayers PM, Groenvold M, Petersen MA, Scott NW, Sprangers MA, Velikova G, Aaronson NK (2016). Group EQoL: replication and validation of higher order models demonstrated that a summary score for the EORTC QLQ-C30 is robust. J Clin Epidemiol.

[11] Gujral S, Conroy T, Fleissner C, Sezer O, King P, Avery K, Sylvester P, Koller M, Sprangers M, Blazeby J (2007). Assessing quality of life in patients with colorectal cancer: an update of the EORTC quality of life questionnaire. Eur J Cancer.

[12] Van Der Hout A, Neijenhuijs KI, Jansen F, van Uden-Kraan CF, Aaronson NK, Groenvold M, Holzner B, Terwee CB, van de Poll-Franse LV, Cuijpers P (2019). Measuring health-related quality of life in colorectal cancer patients: systematic review of measurement properties of the EORTC QLQ-CR29. Support Care Cancer.

[13] Mazanec SR, Sattar A, Delaney CP, Daly BJ (2016). Activation for health management in colorectal cancer survivors and their family caregivers. West J Nurs Res.

[14] Daniele A, Divella R, Abbate I, Casamassima A, Garrisi VM, Savino E, Casamassima P, Ruggieri E, De Luca R (2017). Assessment of nutritional and inflammatory status to determine the prevalence of malnutrition in patients undergoing surgery for colorectal carcinoma. Anticancer Res.

[15] Uhm KE, Yoo JS, Chung SH, Lee JD, Lee I, Kim JI, Lee SK, Nam SJ, Park YH, Lee JY (2017). Effects of exercise intervention in breast cancer patients: is mobile health (mHealth) with pedometer more effective than conventional program using brochure?. Breast Cancer Res Treat.

[16] Rikli RE, Jones CJ (2013). Development and validation of criterion-referenced clinically relevant fitness standards for maintaining physical independence in later years. Gerontologist.

[17] Kampshoff CS, Chinapaw MJ, Brug J, Twisk JW, Schep G, Nijziel MR, van Mechelen W, Buffart LM (2015). Randomized controlled trial of the effects of high intensity and low-to-moderate intensity exercise on physical fitness and fatigue in cancer survivors: results of the resistance and endurance exercise after ChemoTherapy (REACT) study. BMC Med.

[18] Bohannon RW, Wang Y-C, Gershon RC (2015). Two-minute walk test performance by adults 18 to 85 years: normative values, reliability, and responsiveness. Arch Phys Med Rehabil.

[19] Zopfs D, Theurich S, Grosse Hokamp N, Knuever J, Gerecht L, Borggrefe J, Schlaak M (2020). Pinto dos Santos D: single-slice CT measurements allow for accurate assessment of sarcopenia and body composition. Eur Radiol.

[20] Kupsch J, Jackisch T, Matzel KE, Zimmer J, Schreiber A, Sims A, Witzigmann H, Stelzner S (2018). Outcome of bowel function following anterior resection for rectal cancer—an analysis using the low anterior resection syndrome (LARS) score. Int J Colorectal Dis.

[21] Kim CW, Jeong WK, Son GM, Kim IY, Park JW, Jeong S-Y, Park KJ, Lee S-H (2020). Validation of korean version of low anterior resection syndrome score questionnaire. Annals of coloproctology.

[22] Hartrick CT, Kovan JP, Shapiro S (2003). The numeric rating scale for clinical pain measurement: a ratio measure?. Pain Pract.

[23] Norman CD, Skinner HA (2006). eHEALS: the eHealth literacy scale. J Med Internet Res.

[24] Hawkes AL, Chambers SK, Pakenham KI, Patrao TA, Baade PD, Lynch BM, Aitken JF, Meng X, Courneya KS (2013). Effects of a telephone-delivered multiple health behavior change intervention (CanChange) on health and behavioral outcomes in survivors of colorectal cancer: a randomized controlled trial. J Clin Oncol.

[25] Mortazavi BJ, Gutierrez-Osuna R. A Review of Digital Innovations for Diet Monitoring and Precision Nutrition. J Diabetes Sci Technol 2021:19322968211041356. 10.1177/19322968211041356 PMC9846399 34467803

[26] Maisto M, Diana B, Di Tella S, Matamala-Gomez M, Montana JI, Rossetto F, Mavrodiev PA, Cavalera C, Blasi V, Mantovani F et al. Digital Interventions for Psychological Comorbidities in Chronic Diseases-A Systematic Review. J Pers Med 2021, 11(1). 10.3390/jpm11010030 PMC7825345 33418971

